# Computational Fluid Dynamics (CFD) interpretation of backfill pipeline system damage caused by paste-like slurry in water hammers

**DOI:** 10.1371/journal.pone.0310087

**Published:** 2025-08-29

**Authors:** Hao Wang, Yuxin Hao, Zhongmin Ji, Zihan Tang, Ding Pengchu

**Affiliations:** 1 School of Civil Engineering, Zhengzhou University of Technology, Zhengzhou, Henan, China; 2 School of Energy and Mining Engineering, China University of Mining and Technology (Beijing), Beijing, China; 3 School of Civil Engineering, Henan Polytechnic University, Jiaozuo, Henan, China; 4 College of Intelligent Construction and Civil Engineering, Zhongyuan University of Technology, Zhengzhou, Henan, China; NED University of Engineering and Technology, PAKISTAN

## Abstract

Paste-like slurry which made up of gangue particles and fly ash consolidated in the goaf could effectively reduce surface subsidence and improve resource recovery. The technology relies on efficient transport. However, there are many pipeline explosion accidents caused by water hammer due to rapid valve closure. The density and elasticity modulus of the paste-like slurry are significantly greater than other fluids. That makes the pressure exerted on the pipeline and valve by the slurry water hammer is significantly greater than the other fluids. The research established the relationship between valve closing time and peak pressure using dynamic meshes combining theoretical analysis and numerical simulations. The results show that the velocity at which the valve closes significantly affects the pressure on the valve and nearby pipelines. However, when the valve large open, the impact only on the local flow velocity and has no substantial impact on the flow state throughout the system. Finally, the optimal closing time of the butterfly valve was determined by comparing pressure magnitudes.. The paper focuses on the phenomenon of water hammer in dense slurries, which is different from other fluids. However, theoretical and simulation models have reference value for the study of other fluid water hammer.

## Introduction

The solid materials hydraulic transporting is widely used. In coal backfill mining, the backfill slurry is transported into goaf by pipelines. The high pressure caused by water hammers will occur several times, and the periodic change may damage the pipeline and its auxiliary facilities. At the end of the nineteenth century, researchers such as Weston [1], Frizell [2] attempted to explain the relationship between pressure and velocity changes. Joukowsky [3] developed the famous “fundamental equation of the water hammer” in transient flow theory. Korteweg [4] proposed equations to account for the geometric properties of the pipe and other fluid materials, as well as the elastic modulus. Chaudhry [5], Wylie [6] and Lighthill et al. [7] established a water hammer velocity equation for compressible fluids in flexible pipelines. Zhou et al. [8] presented mathematical and numerical modeling technology used for simulated transient pressure in the abnormal pump operation. The results showed negative correlation between volume concentration and maximum transient pressure, the period numbers of pressure decay, and the corresponding shock wave speed. Wang et al. [9] reported the pulse and pulsating supercharging phenomenon induced by the internal pressure wave in semi-closed pipe. The results show that a remarkable supercharging phenomenon exists at the pipe end face, the supercharging effect is very significant at the family of optimal frequencies.

The paste-like slurry moves in the pipeline as a “plunger flow” and could be considered as high-density homogeneous slurry when calculating the water hammer pressure and speed. If the valve closes instantly, the velocity of the slurry adjacent to the valve immediately decreases to *0*. Due to the effect of inertia, the rest of the slurry continues to move through the pipeline. The squeezed slurry compresses and deforms, generating additional pressure and propagating as a pressure wave in the direction of the slurry flow. As a result, the pressure near the valve and the surrounding pipeline increases sharply. If the pressure exceeds the limit of the valve or pipeline, an explosion will occur. Han et al. [10] deduced continuity equations and momentum equations of pseudo-homogeneous flows, and built a pseudo-homogeneous water hammer model. The model considered characteristics of solid-liquid flow’s viscosity, resistance and wave velocity. Norouzi et al. [11] studied the fluid hammer of viscoelastic flow in pipes by the Oldroyd-B model which used as the constitutive equation. The results show that the attenuation of the laminar fluid transient is affected by viscoelastic properties of the non-Newtonian fluid. The above researches show that, unsteady multiphase and multicomponent flows in closed pipe are common in practice. Because the bulk modulus and density of the mixture are different from water, the wave velocity and pressure influenced by the presence of these phases and components are differently.

The fixed-grid MOC method has been used to calculate both the pressure and velocity fields in pipe systems and networks under transient conditions. In practice, because pipes have different lengths, it is impossible to exactly satisfy the common time step required by the fixed-grid MOC used to solve the governing equations in all pipes. To address this discretization problem, researchers have tried a variety of methods, such as the use of linear space-line interpolation to approximate heads and flows at the bottom of each characteristic line; the use of different time steps for each pipe [12]; and the combination of traditional space-line interpolation with reach-out in space interpolation [13]. Saffar et al. [14] used finite element analysis to study the effect of water hammers on the damage area of steel pipes and the behavior of composite patches in repairing steel pipe damage. Cao et al. [15] adopted the MOC to analyze the velocities and dynamic pressures of fracturing fluid, as well as the axial velocities and additional stresses in pipes at different depths. On this basis, they quantitatively analyzed the water hammer effect and vibration characteristics during pump starting and stopping. Moosavian [16] proposed a matrix equation that differed from the traditional MOC method; this technique was used to simultaneously solve for all the pressures and flows in the network at each time step as a linear function of the pressure and flow at all points in the network at the previous time step.

It is difficult to calculate the water hammer pressure and propagation wave velocity by nonlinear motion and continuity equation directly. Apoloniusz [17] described and analyzed the results of physical experiments with a water hammer in steel and PE pipelines. Chen et al. [18] established four-way direction control valve models for butterfly valves and ball valves. These ingenious and practical methods have greatly accelerated research on water hammers. Kahraman [19] studied the optimum sudden shutdown time for the adjusting wings in a snail based on the impact of the water hammer pressure and the turbine shaft overspeed under various hydroelectric power plant operating conditions. Due to the distinct densities and elastic moduli, the pressures formed by the slurry and water in the water hammer differ. Jiang et al. [20] compared Kagawa model and impulse response function model in the describe of the dynamic friction force. It is shown that the result of impulse response function model is closer than Kagawa’s model to the experimental curves for transient pressure estimation.

The goal of computer-aided calculate and analysis is to improve humans´ understanding of complex physical phenomena and the ability of predict and control these phenomena. The various physical models and advanced numerical methods available in FLUENT could help researchers in determining the performance associated with different coefficients to predict the torque, flow rate, and accompanying flow field of butterfly valves. The dynamic mesh method of CFD is an efficient tool for describing the nonlinear flow of water hammers. The dynamic mesh method combines the *Lagrangian* and *Eulerian* methods; thus, the mesh can move at any velocity when solving the unsteady flow caused by changes in the calculation domain. Chattopadhyay et al. [21] investigated the turbulent flow structure inside a PSROV using ANSYS-FLUENT software and discussed critical features, including compressibility and turbulence. With the dynamic mesh technique and a CFD solver, Srikanth and Bhasker [22] studied compressible air flows in a typical puffer chamber. Shih et al. [23] proposed a realizable *k–e* model by solving the time-averaged momentum equation. This model was able to satisfactorily compute the turbulent viscosity term. Paste-like slurry is a typical multiphase flow, and its behavior in a pipeline is complex and difficult to predict [24]. The multiphase flow model of commercial CFD software is an effective method for handling pipeline transportation problems. Zhou et al. [25] used elastic smoothing and local mesh redrawing to dynamically simulate the valve closing process and calculated the slurry water hammer pressure caused by rapid valve closure. Liu et al. [26] studied the slurry flow field in four valves with numerical methods, physical models, the dynamic mesh method and user-defined functions (UDF) in FLUENT. The results revealed that the loss increases as the opening of the valve decreases and that the processes of opening and closing the valve are not simply reverse processes. Zhou et al. [27] studied the slurry water hammer pressure of a solid–liquid two-phase flow by using spring-base smoothing technology and local remeshing technology with a dynamic mesh and CFD. The results showed that the maximum pressure in the pipeline was negatively correlated with the valve closing time and positively correlated with the initial flow velocity. Joshi et al. [28,29] proposed that the slurry efficient transporting must be at low velocity and high Prandtl number, the result based on the numerical simulations for slurry flow through a horizontal pipeline at different roughness heights and Prandtl number.

The mass concentration of the gangue-fly ash filled slurry was approximately 78%, indicating that it had the physical properties of a Bingham body, in particular, the maximum particle size was approximately *20* mm. In engineering, there are often scenarios of staged backfilling, such as strip backfill mining. In the situation, the backfill system needs to be closed temporarily, resulting in “slurry water hammer” phenomenon. Backfill slurry is a dense fluid, the flow characteristics cannot be simply imitated from other fluids for significantly different density and rheological parameters. Research on paste-like backfill slurry in water hammers is lacking. This paper combines the method of MOC and FLUENT simulation to solve the velocity and pressure during the water hammer process of dense slurry. Thus, it would be interesting to perform CFD simulations on this kind of slurry in a water hammer, especially by introducing dynamic mesh method into the simulation process, it will more in line with the real situation. However, theoretical and simulation models have reference value for the study of other fluid water hammer.

## Theoretical analysis

### Viscoelastic plastic effect of slurry and its role in water hammers

**Fig 1:**
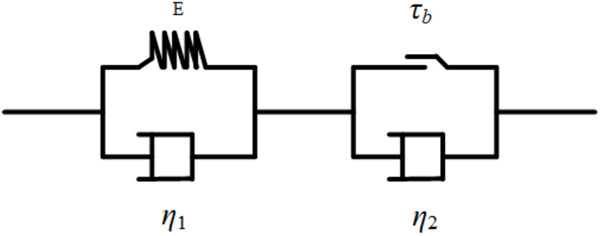
Viscoelastic plastic model diagram of backfill slurry.

Slurry contains fly ash, cement and gangue particles. Because the coarse and fine particles are mixed together, the slurry is viscous. When the external shear force is greater than the initial shear stress, the slurry exhibits both elastic deformation and irreversible plastic flow characteristics. However, in some cases, the slurry may exhibit only elastic deformation characteristics. Elastic deformation does not occur and immediately recovers after the external force is removed due to the viscous effect. In summary, the gangue-fly ash filled slurry shows the characteristics of a viscoelastic solid before yielding and those of a viscoelastic plastic body after yielding. The slurry is viscous not only in the elastic state before yielding but also in the plastic flow state after yielding. Diagram of viscoelasticity representing the physical properties of backfill slurry is shown as Fig 1.

The meaning of the symbols in Fig 1 are as following:

*E*: Modulus of elasticity of the slurry;

*τ*_*b*_: Initial yield stress of the slurry;

*η*_*1*_: Elastic viscosity of the slurry under dynamic conditions;

*η*_*2*_: Plastic viscosity of the slurry in steady flow.

Due to the dual action of the elastic effect of the backfill slurry and water hammer phenomenon, the pipeline periodically expands, deforms and can even rupture. The elastic characteristic can also provide resistance calculations and parameter optimization for the design of paste-like filling slurry transportation pipelines. Therefore, the elastic characteristic of gangue-fly ash backfill slurry is an important physical and mechanical property that needs to be fully considered in rheological property research.

The previous research developed an intelligent torque rheometer for testing coarse particle slurries and carried out several experiments. The results shown that the mass concentration of the slurry must remain below 78% and that the optimal slurry composition includes coal gangue, fly ash, and a gelling agent at a weight ratio of 8:3:1. The viscosity coefficient of this slurry is 2.67 *Pa·s*, and the initial yield stress is 101.7 *Pa*. The slurry parameters used in this paper are taken from the above studies [30,31].

### MOC (Methods of Characteristic) calculation of the pressure and wave speed of the filling slurry in the water hammer

The pressure in the valve peaks after the first shock wave ends. According to the momentum theorem [28,29]. As equation (1) and (2):


(A+ΔA)ΔpΔt=MΔU (1)

$$\Delta p=\frac{\rho \Delta UL}{\Delta t}$$ (2)

ρ: Density of the slurry, *kg/m*^*3*^;

A: Original cross-sectional acreage of the pipeline;

Δp: Additional pressure in water hammer;

ΔU: Difference between the initial and final flow rates of the slurry;

ΔU=U, in a direct water hammer;

Δt: Time between valve closure and the first peak.

The change in the slurry volume is equal to the sum of the pipe volume expansion, and the slurry is compressed regardless of the change in the pipe length. As equation (3):


$$\Delta V=\Delta V_{p}+\Delta V_{F}=A\Delta U\frac{\Delta L}{\alpha }$$

$$\Delta V_{p}=\Delta LA\frac{pD}{E_{p}e}$$

$$\Delta V_{F}=\frac{pA\Delta L}{E_{F}}$$

$$A\Delta U\frac{\Delta L}{\alpha }=\Delta LA\frac{pD}{E_{p}e}+\frac{pA\Delta L}{E_{F}}$$ (3)

$\Delta V_{p}$: Increase in the pipe volume;

$\Delta V_{F}$: Compression of the slurry volume;

A: Original cross-sectional acreage of the pipeline;

ΔL: Length of the pipeline;

*p*: Relative water head of the point in the pipe, *m*;

*E*_*p*_: Elastic modulus of the pipeline;

*E*_*F*_: Elastic modulus of the slurry;D: Inner diameter of the pipe.

The shock wave propagation velocity of the filling slurry differs from that of water because of the large amount of gangue particles in the slurry. As equation (4):


$$\alpha_{m}=\sqrt{\frac{\frac{E_{h}}{\rho }}{1-C_{QV}+\frac{E_{w}}{E_{s}}C_{QV}+\frac{E_{h}D}{E_{p}e}}}$$ (4)

$\alpha_{m}$: Velocity of the pressure wave;

$C_{QV}$: Volume concentration of the gangue particles;

$E_{p}$: Elastic modulus of the pipe;

*e*: Pipeline wall thickness;

$E_{w}$: Elastic modulus of the slurry;

ρ: Slurry density;

$E_{s}$: Elastic modulus of the gangue particles.

The governing equations and boundary conditions of the indirect water hammer are nonlinear equations and are thus difficult to solve with analytical methods. The method of characteristics is effective for solving such problems because the governing equations of an indirect water hammer can be transformed into hyperbolic equations. The principle of the characteristic method is to convert the partial differential equations into ordinary differential equations in the characteristic direction, i.e., to convert the characteristic equations, and then convert the equations into a first-order finite difference scheme to obtain the approximate solution.

The governing equations of the indirect water hammer are as equation (5) and (6):


$$\text{Motion\textbackslash{} equation}:\frac{\partial P}{\partial x}+\frac{v}{g}\frac{\partial v}{\partial x}+\frac{1}{g}\frac{\partial v}{\partial x}+\frac{\partial L}{\partial x}+\frac{2\tau }{\rho gR}=0$$ (5)

$$\text{Continuity\textbackslash{} equation}:\frac{\partial P}{\partial t}+v\frac{\partial P}{\partial x}+\frac{U^{2}}{g}\frac{\partial v}{\partial x}=0$$ (6)

P: Relative water head of the point in the pipe, *m*;

g: Gravitational acceleration, *m/s*^*2*^;

x: Distance from the point to the valve;

f: Drag coefficient along the pipe;

U: Wave velocity of the water hammer, *m/s*;

L: Length of the pipe;

v: Velocity of the slurry;

t: Flow time, *s*.

To simplify the calculations based on the following assumptions: the change in the horizontal pipe diameter was assumed to be negligible, and the slurry flow velocity was assumed to be negligible relative to the pressure wave velocity. The simplified equations of motion and continuous equations can be described as equation (7) and (8):


$$\text{Equation\textbackslash{} of\textbackslash{} motion}:g\frac{\partial P}{\partial x}+\frac{\partial v}{\partial t}+\frac{f}{2gD}v|v|+\Delta F=0$$ (7)

$$\text{Continuity\textbackslash{} equation}:\frac{\partial P}{\partial t}+\frac{u^{2}}{g}\frac{\partial U}{\partial x}=0$$ (8)

The ordinary differential equations converted from (1) and (2) along the characteristic curve are as equation (9) and (10):


$$\text{C}+:\left\{ \begin{array}{c} \frac{dV}{dt}+\frac{g}{c}\frac{dH}{dt}+\frac{fV|V|}{2D}=0\\ \frac{dx}{dt}=+a_{m} \end{array}\right.$$ (9)

$$\text{C}-:\left\{ \begin{array}{c} \frac{dV}{dt}-\frac{g}{c}\frac{dH}{dt}+\frac{fV|V|}{2D}=0\\ \frac{dx}{dt}=-a_{m} \end{array}\right.$$ (10)

The total derivative term can be discretized into equation (11) and (12) with the differential method:


$$\text{C}+\;:\left\{ \begin{array}{c} H_{i}=H_{i-1}-\frac{a_{m}}{gA}\left({Av}_{p}-{Av}_{i-1}\right)-\frac{f_{m}a_{m}\Delta t}{2gD}v_{i-1}\left|v_{i-1}\right|\\ x_{i}-x_{i-1}=a_{m}(\Delta t) \end{array}\right.$$ (11)

$$\text{C}-:\left\{ \begin{array}{c} H_{i}=H_{i-1}+\frac{a_{m}}{gA}\left({Av}_{i}-{Av}_{i-1}\right)+\frac{f_{m}a_{m}\Delta t}{2gD}v_{i-1}\left|v_{i-1}\right|\\ x_{i}-x_{i-1}=-a_{m}(\Delta t) \end{array}\right.$$ (12)

The solution to the above equations is shown as equation (13) and (14):


$$Q_{i}=\frac{gA[H_{i}+Q_{i}(\frac{a_{m}}{gA}-\frac{fa_{m}\Delta t}{2gDA^{2}}\left|Av_{i}\right|)-C_{m}]}{2a_{m}}$$ (13)

$$H_{i}=\frac{H_{i}+Q_{i}(\frac{a_{m}}{gA}-\frac{fa_{m}\Delta t}{2gDA^{2}}\left|Av_{i}\right|)-C_{m}}{2}$$ (14)

The equation can be solved by the recursive method according to the above formulas. At the beginning, the initially mesh values were determined, then, calculate the values of adjacent mesh, and repeat the process to obtain the values of the specified mesh.

## Numerical simulation

### Meshing

**Fig 2:**
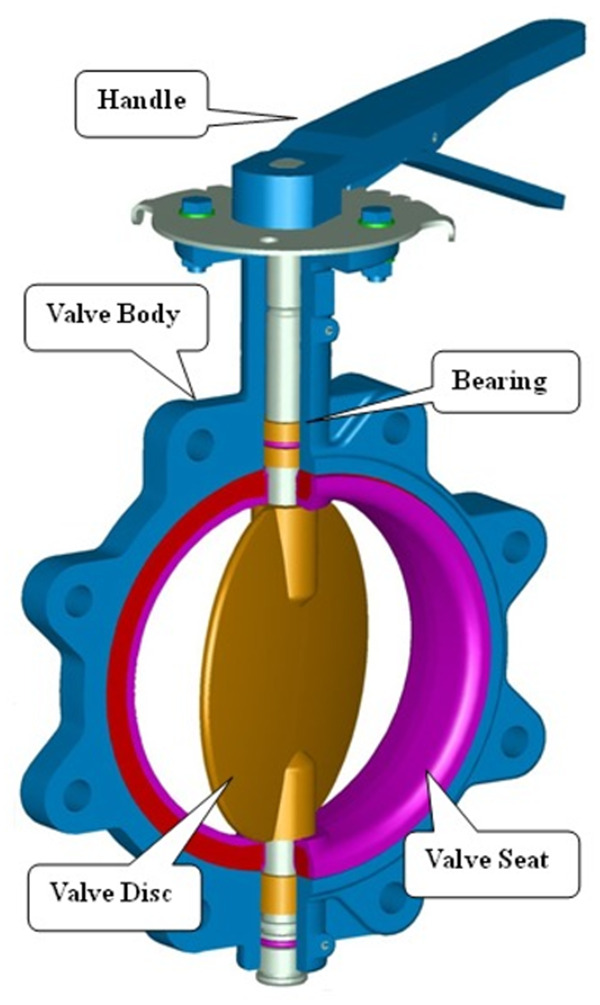
The diagram of the butterfly valve.

The extension of the closure time is one of the most commonly used methods for reducing the maximum pressure increase [32,33]. In this paper, the relationship between the pressure of the water hammer and the valve closing time was investigated. When the valve closes, the boundary of the mesh model changes continuously. During this process, the pipeline is suffered dynamic pressure due to the dynamic state of the slurry. Thus, dynamic mesh technology is suitable for addressing this problem. The main idea of dynamic mesh technology is to update the flow field mesh at each time step based on the changes in the computational domain due to boundary changes. The mesh update process is automatically performed by FLUENT software based on changes in the boundaries at each iteration step. A diagram of the butterfly valve is shown in Fig 2.

**Fig 3:**
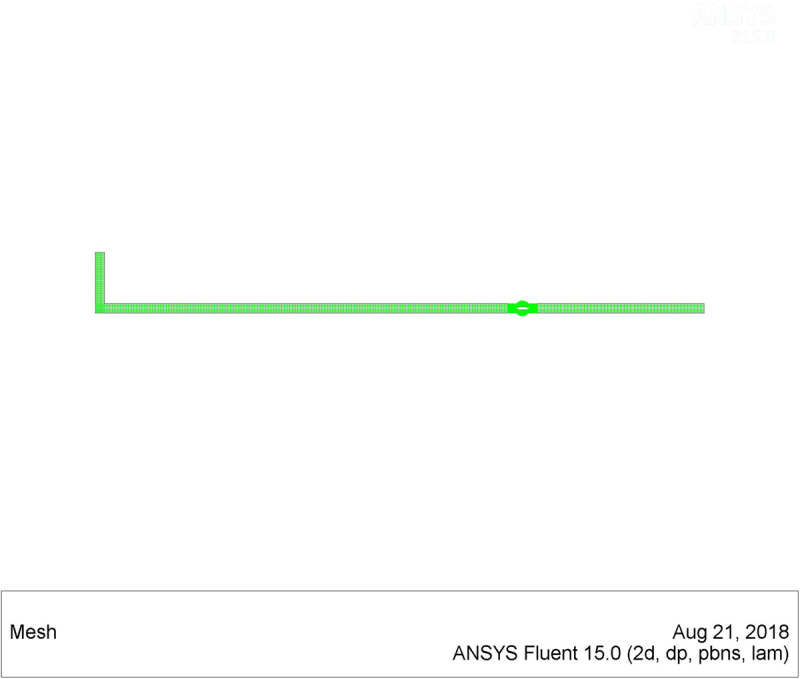
Schematic diagram of the pipeline mesh (valve part).

**Fig 4:**
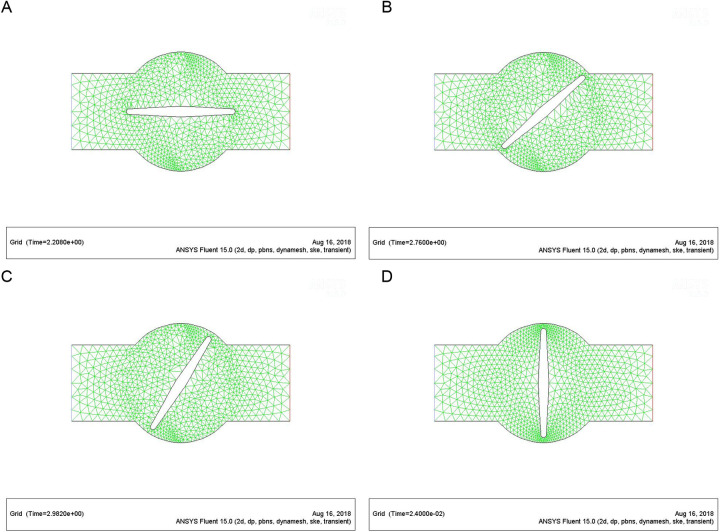
(a). Initial state enlargement diagram of the valve (valve part). (b). Mesh of the valve rotating at 60° (valve part). (c). Mesh of the valve rotating at 60° (valve part). (d). Mesh of the closed valve (valve part).

The pipeline model had a length of 3,000 mm and a diameter of 150 mm. There were 1.012 million unstructured meshes in total. There should be at least three meshes between the valve disc and the pipe seat to prevent accuracy errors during the rotation of the valve. Dynamic mesh was used to make simulates more realistic. The two-dimensional mesh schematic diagrams at four angles (0 °, 45 °, 60 °, 90 °) during the valve disc rotating were displayed in Fig 3–Fig 4.

### Boundary condition setting

**Fig 5:**
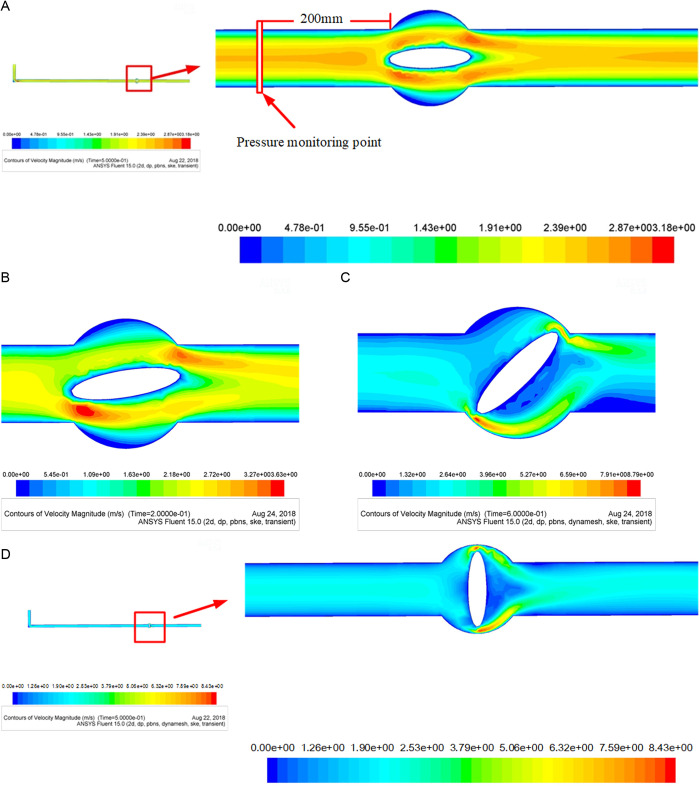
(a) Schematic diagram of the velocity distribution (fully open). (b) Schematic diagram of the velocity distribution near the valve disc (rotating at π/20). (c) Schematic diagram of velocity distribution near the valve disc (rotating at π/4). (d) Schematic diagram of velocity distribution (fully closed).

The valve was actively closed, with the closing rules and speeds defined by the valve profile and closing times of 1 s, 2 s, 3 s, and 4 s. Consider the cross section at a distance of 0.2 m from the left side of the valve as the object. The results show the different water hammer pressures at different closing velocities. The slurry density is 2000 kg/m^3^, the inlet velocity is 1.4m/s, outlet pressure is 20000 Pa; The mixed slurry of fly ash and cement was set as the fluid phase, and gangue particles were set as the dense discrete phase. The particle size of gangue particles was set to follow a normal distribution, with particles with a diameter of 20 mm accounting for 10%. Considering the settlement of gangue particles, the error of exported and input gangue particles number is below 5%. The valve disc begins to move after 2 seconds as the initial value under transient algorithms. And the motion state of the slurry in the pipeline system begins to change. When the valve disc moves downward to near complete closure, it stops moving and closes the dynamic mesh. The calculation continues until the pulsating pressure is eliminated. The velocity distributions for valve fully opening degrees are shown in Fig 5(a) (Fig 5(a).).

In this process, the valve disc does not contact the pipe seat fully to ensure that the slurry flows in the pipe and that the FLUENT software continued its calculations.

The figures show that when the valve is fully opened, the slurry in the pipe section before the valve was essentially in a laminar flow state with a clear flow nucleus. As the slurry moves, the scale of the flow nucleus clearly increases because of the obstruction of the valve before splitting into two parts. The average velocity of the slurry at the ends of the valve is greater than the original velocity, and the velocity at the bottom of the valve is greater than the velocity at the top of the valve. The flow has a velocity of zero near the outer spherical face of the valve, and the slurry depositing because of gravity, especially near the lower spherical face of the vale. The maximum velocity occurs at the top of the valve, and the slurry transitions back to laminar flow with a visible nucleus beyond the valve. Thus, the valve has an impact only on the local flow state of the slurry and has no substantial impact on the flow state of the slurry throughout the system.

Fig 5(b) (Fig 5(b)) shows that when the valve rotates at π/20, the distance between the valve disc and valve seat is reduced. According to the conservation of mass law, the flow rate increases as the cross-sectional area decreases. The flow rate of the slurry near the ends of the valve is significantly larger than the original flow rate, and the velocity at the bottom of the valve is greater than the velocity at the top of the valve. The velocity is always zero near the outer spherical wall of the valve.

The valve is nearly closed after it is rotated by π/4, and the velocity of the slurry is nearly zero. As shown in Fig 5(c) (Fig 5(c)), the velocity near the bottom of the valve disc increases, while the velocity at the top of the valve remains zero.

Fig 5(d) (Fig 5(d)) shows that as the valve closes, the area near the back of the valve where the flow velocity is nearly 0 increases. When the valve is fully closed, the slurry in the pipeline system stops flowing.

In short, the velocity of the slurry changes significantly when the valve closes. Moreover, the area of high velocity between the valve and the pipe wall decreases sharply, and the maximum velocity increases significantly. The area of high velocity near the lower end of the valve is always larger than that near the upper end of the valve. There is a certain velocity “blind zone” behind the valve disc where the velocity of the slurry is nearly 0; the slurry sediments in this region. After the valve closes, the “blind area” decreases until the closing angle of the valve reaches π/4, at which point the “blind area” essentially disappears, indicating that the slurry flow structure is disrupted by the valve and the flow direction has changed. Furthermore, the velocity of the slurry in the pipe adjacent to the valve is 0. This area increases as the valve closes. This area reaches its maximum when the closing angle of the valve reaches π/4; then, the area gradually disappears. The area near the lower wall where the velocity is 0 is significantly larger than the corresponding area near the upper wall.

### Pressure conditions under different valve opening degrees

**Fig 6:**
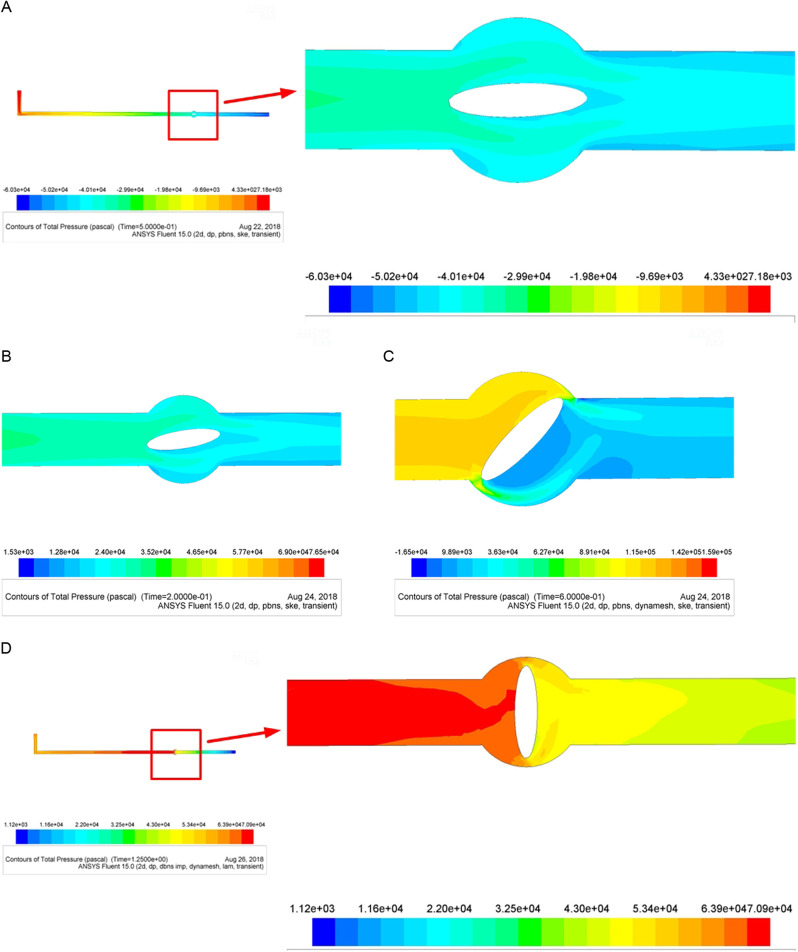
(a) Schematic diagram of the pressure condition when the valve is fully open. (b) Schematic diagram of the pressure distribution near the valve disc (rotating at π/20). (c) Schematic diagram of the pressure distribution near the valve disc (rotating at π/4). (d) Schematic diagram of the pressure distribution when the valve is fully closed.

Fig 6(a) (Fig 6(a)) shows that as the pipeline extends, the total pressure of the slurry decreases. The pressure distributions near the upper and lower sides of the valve are essentially symmetrical, and a low-pressure area with a length of distance appears on the pipe wall at the rear end of the valve.

Fig 6(b) shows when the rotation angle is π/20, the pressure near the valve is essentially the same as when the valve is in its initial state. There is a small low-pressure area only near the lower half of the valve wall, which has little effect on the slurry flow state.

Fig 6(c) shows the pressure near the valve changes significantly when the rotation angle is π/4. The slurry stops suddenly near the front of the valve, but the slurry input is continuous. Thus, the slurry will deform due to valve blockage, and then will convert into elastic potential energy which is converted into a large and instant pressure on the pipe and valve.

Fig 6(d) shows that the pressure distribution is divided by the valve. The inlet direction of the valve is a high-pressure zone, while the outlet direction of the valve is a low-pressure zone. As the distance from the valve increases, the pressure decreases.

### Maximum pressure in the water hammer for different valve closing times

**Fig 7:**
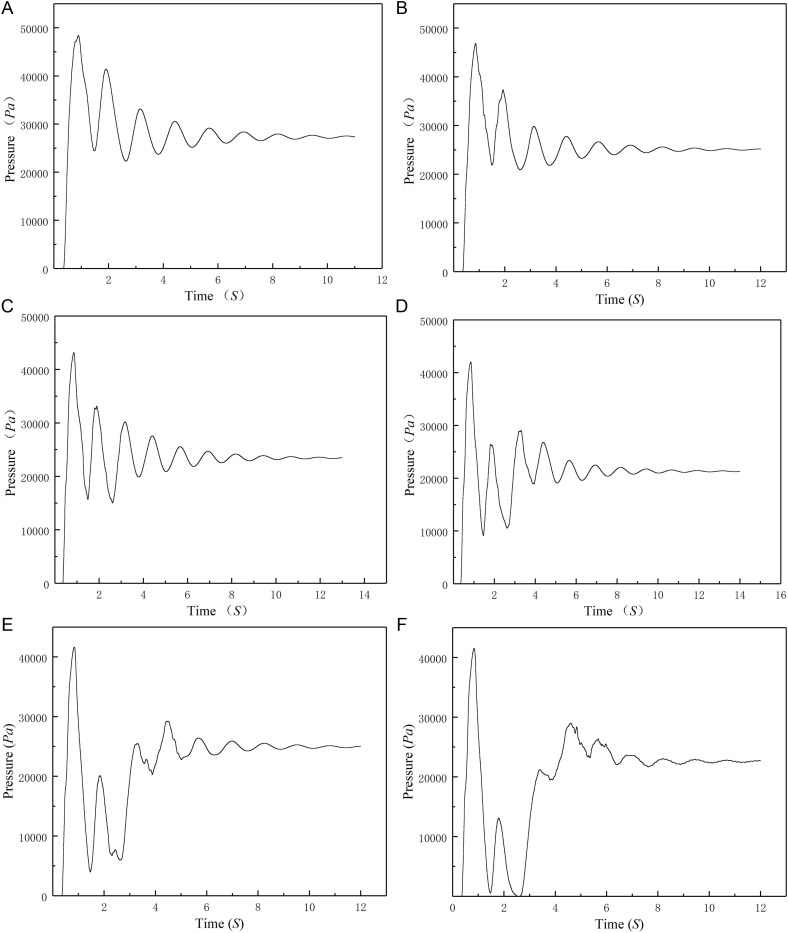
(a) Curve of the pressure state when the valve closes in 1 second. (b) Curve of the pressure state when the valve closes in 2 seconds. (c) Curve of the pressure state when the valve closes in 3 seconds. (d) Curve of the pressure state when the valve closes in 4 seconds. (e)Curve of the pressure state when the valve closes in 5 seconds. (f) Curve of the pressure state when the valve closes in 6 seconds.

Fig 7(a) (Fig 7(a)) shows the pressure state when the valve closes in 1 second.

Fig 7(b) (Fig7(b)) shows the pressure state when the valve closes in 2 seconds. Fig 7(c) (Fig 7(c)) shows the pressure state when the valve closes in 3 seconds. Fig 7(d) (Fig7(d)) shows the pressure state when the valve closes in 4 seconds. Fig 7(e) (Fig 7(e)) shows the pressure state when the valve closes in 5 seconds. Fig 7(f) (Fig 7(f)) shows the pressure state when the valve closes in 6 seconds.

As seen from Fig 7, the average total pressure of the pipe sections decreases with the valve closing time. The total pressure of the section is just the pressure when the valve is fully opened. When the valve rotation arc is π/20, the pressure reaches its minimum; when the valve rotation arc is π/4, the pressure reaches its maximum and remains in this high-pressure state until the valve is closed. The maximum pressure appears many times. All the pressure curves are very similar and do not vary depending on the valve closing time.

## Results

**Fig 8:**
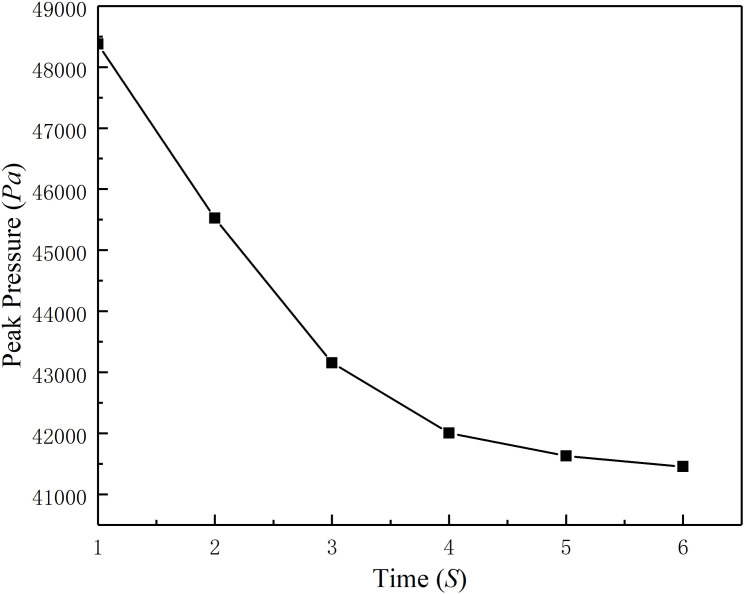
Curve of the peak pressure versus the valve closing time.

(1) When the valve is fully open, the slurry is keeps at laminar flow state in the horizontal pipeline. The mixture of gangue particles and slurry moved together without obvious separation, showing good mobility. As the valve closed, the flow cross-sectional area decreased, and part static pressure transformed into dynamic pressure, causing an increase in slurry flow velocity near the valve. Regardless of the valve opening, the pressure at the back end of the valve is significantly lower than that at the front end, indicating that the valve has a significant hindering effect on fluid flow, resulting in a much lower static pressure of the fluid passing through the valve than the static pressure at the inlet. This indicates that valves have a significant impact on flow.
(2) As the valve opening decreases until completely closed, the inlet velocity of the slurry decreases and eventually drops to 0. The phenomenon is in line with reality.
(3) Fig 8 (Fig 8) shows the peak pressure versus the valve closing time. The peak pressure of the slurry water hammer decreases rapidly as the valve closing time increases. As the closing time increases, the peak pressure formed by water hammer in the slurry decreases significantly. When the valve closing time is less than 3 s, the peak pressure clearly decreases; when the valve closing time is between 3 s and 4 s, the pressure decreases slightly; and when the valve closing time is greater than 4 s, the pressure drop gradually decreases, indicating that if the valve closing time is greater than 4 s, its effect on the peak pressure drop is limited. Therefore, the valve closing time should be at least 4 s.

## Discussion

The wall thickness of the slurry filled pipeline can be calculated based on strength theory just as equation (15) and (16) [34,35]:


$$h=\frac{k\cdot p\cdot D}{2[\delta ]\cdot E\cdot F}+C_{1}T+C_{2}$$ (15)

$$p=\frac{2{\left[\delta \right]}_{1}\cdot E\cdot F\cdot (h+C_{1}T+C_{2})}{k\cdot D}$$ (16)

D: Minimum diameter;

H: Thickness of the pipe wall;

p: Maximum working pressure of the pipeline;

[δ]: Tensile strength of the pipe, MPa (usually 80% of the minimum yield stress);

E: Coefficient of weld;

F: Regional factor;

T: Service time, a;

$C_{1}$: Wear rate in one year, mm/a;

$C_{2}$: Additional thickness, mm;

k: Coefficient of pressure.

The wall thickness of the pipe changes from $h_{1}$ to $h_{2}$ due to the extrusion of the slurry in the water hammer.

The equation (17) and equation (18) established according to the principle of conservation of volume:


$$\pi {\left(r+h_{1}\right)}^{2}\cdot L-\pi r^{2}\cdot L=\pi {\left(r+h_{1}+h_{2}\right)}^{2}\cdot L-\pi {\left(r+h_{1}\right)}^{2}\cdot L$$ (17)

$$h_{2}=\sqrt{2{\left(r+h_{1}\right)}^{2}-r^{2}}-r-h_{1}$$ (18)

The maximum allowable pressure changes because the wall of the pipe becomes thinner. This change can be calculated by equation (19):


$$p_{2}=\frac{2{\left[\delta \right]}_{2}\cdot E\cdot F\cdot (h_{2}+C_{1}T+C_{2})}{k\cdot D}$$ (19)

The equation (20) describes ratio of the ultimate pressure that the pipeline can withstand before and after the water hammer:


$$\lambda =\frac{p_{1}}{p_{2}}=\frac{{\left[\delta \right]}_{2}(h_{2}+C_{1}T+C_{2})}{{\left[\delta \right]}_{1}(h_{1}+C_{1}T+C_{2})}$$ (20)

The above formulas show that the change in the maximum working pressure of the pipeline depends on the variation in the tensile strength of the pipeline and the thickness of the pipeline wall. Furthermore, the service life of the pipeline has a certain impact on the ratio of the pressure before and after the water hammer. To ensure the normal operation of the pipeline system, some safety redundancy should be introduced, and the valve closing time should be extended.

## Conclusions

Water hammer is a common phenomenon in fluid pipeline transportation. The water hammer of dense slurry often causes more serious result. The local pressure on the wall of the pipe (especially near the valve) increases sharply in the water hammer, which can lead to pipe rupture and accidents. Over time, the strength of a pipeline is continuously reduced due to slurry wear and impact.

(1) In this paper, the water hammer in a slurry pipeline system was analyzed, and the wave velocity and pressure of direct and indirect water hammers were calculated independently based on the MOC theory.
(2) The paper described the effect of the water hammer on the allowable working pressure of the filled pipeline based on the Unified strength theory. The ultimate pressure of the pipeline changed due to variations in the tensile strength and thickness of the pipe wall in the water hammer. That means water hammer will cause accidents more likely as the service life of pipelines increases.
(3) The state of the slurry in the pipe changed with the rotation of the valve. Therefore, the dynamic mesh method can be used to intuitively visualize the state of the slurry in the water hammer. The simulation results show that, when the valve is not fully closed, the valve slowly closes has an impact only on the local flow velocity of the slurry and has no substantial impact on the flow state of the slurry throughout the system. But the pressure on the pipeline near the valve will increase.
(4) As the closing velocity of the valve accelerates, the pressure applied on the pipeline near the valve increases rapidly. When the valve closing time is less than 4 seconds, the peak pressure change is strongly correlated with the closing time; if the closing time exceeds 4 seconds, formed a gentle pressure increase trend. Comparing the valve closing time of 4 seconds and 1 second, the gap of peak pressure applied on the pipeline close to 50%. The analysis of the pressure condition near the valve versus the valve closing time showed that the valve closing time should be increased as much as possible, and that the closing time should be at least 4 s.
